# Epigenomic Modifications in Modern and Ancient Genomes

**DOI:** 10.3390/genes13020178

**Published:** 2022-01-20

**Authors:** Laura Niiranen, Dawid Leciej, Hanna Edlund, Carolina Bernhardsson, Magdalena Fraser, Federico Sánchez Quinto, Karl-Heinz Herzig, Mattias Jakobsson, Jarosław Walkowiak, Olaf Thalmann

**Affiliations:** 1 Research Unit of Biomedicine, Faculty of Medicine, University of Oulu, Medical Research Center, Oulu University Hospital, P.O. Box 5000, FIN-90014 Oulu, Finland; laura.niiranen@oulu.fi (L.N.); karl-heinz.herzig@oulu.fi (K.-H.H.); 2 Department of Pediatric Gastroenterology and Metabolic Diseases, Poznań University of Medical Sciences, Szpitalna 27/33, 60-572 Poznan, Poland; leciejdawid@gmail.com (D.L.); jarwalk@ump.edu.pl (J.W.); 3 Laboratory of Molecular Diagnostics Genmed, Sw. Marcin 49, 61-806 Poznan, Poland; 4 Human Evolution, Department of Organismal Biology, Uppsala University, Norbyvägen 18c, 75236 Uppsala, Sweden; hanna.edlund@ebc.uu.se (H.E.); carolina.bernhardsson@ebc.uu.se (C.B.); Magdalena.fraser@ebc.uu.se (M.F.); federicosq@gmail.com (F.S.Q.); mattias.jakobsson@ebc.uu.se (M.J.); 5 Department of Archaeology and Ancient History, Uppsala University-Campus Gotland, Cramérgatan 3, 62157 Visby, Sweden; 6 Instituto Nacional de Medicina Genómica, Mexico City 14610, Mexico; 7 Paleogenomics Group, Faculty of Veterinary Medicine, Ludwig Maximilian University Munich, Kaulbachstr. 37 III, 80539 München, Germany

**Keywords:** epigenetics, DNA methylation, ancient DNA, paleoepigenomics, diet, lifestyle diseases

## Abstract

Epigenetic changes have been identified as a major driver of fundamental metabolic pathways. More specifically, the importance of epigenetic regulatory mechanisms for biological processes like speciation and embryogenesis has been well documented and revealed the direct link between epigenetic modifications and various diseases. In this review, we focus on epigenetic changes in animals with special attention on human DNA methylation utilizing ancient and modern genomes. Acknowledging the latest developments in ancient DNA research, we further discuss paleoepigenomic approaches as the only means to infer epigenetic changes in the past. Investigating genome-wide methylation patterns of ancient humans may ultimately yield in a more comprehensive understanding of how our ancestors have adapted to the changing environment, and modified their lifestyles accordingly. We discuss the difficulties of working with ancient DNA in particular utilizing paleoepigenomic approaches, and assess new paleoepigenomic data, which might be helpful in future studies.

## 1. Introduction

While genetics defines the study of DNA sequences and the effects of associated changes in nucleotide composition, epigenetics focuses on molecular modifications that do not alter the DNA sequence per se, but contribute to variation in phenotypes by affecting, for instance, gene expression. This mechanism is based on a multifaceted regulation of transcriptional activity, such as chromatin structure modulations [1,2]; histone positioning [3,4]; chemical modifications of histones; regulation of transcription factor recruitment [5,6]; and chemical modification of the DNA, i.e., methylation [7]. Selectively important mutations can be introduced and manifested in a population, and hence affect nucleotide composition in a longer term; epigenetic changes, however, are more flexible and can enable the organism to quickly respond to environmental conditions [8,9,10,11,12]. As an example, studies focusing on populations introduced to new habitats suggest that epigenetic changes can compensate for decreased genetic variation due to founder effects, and thereby maintain a degree of phenotypic plasticity [8,9,10,13].

Rapid technological developments in molecular biology now allow for investigations into epigenetic changes at unprecedented depths, and are no longer limited to contemporary specimens [14,15,16,17]. The study of ancient specimens is termed paleoepigenetics [18] (or, to better reflect the omics-era, paleoepigenomics) and has already unraveled intriguing findings that advanced our understanding of the evolutionary history of our very own species [19,20,21,22,23,24,25].

In this review, we discuss epigenetic mechanisms (microRNA, histone modifications, and DNA methylation) and their evolutionary and health implications, and provide an exemplary data set generated from three ancient genomes using the latest paleoepigenomic tools.

## 2. Epigenetic Mechanisms

Epigenetic mechanisms are critically important genetic mediators and are responsible for gene control independent of sequence variation. Understanding the background of epigenetic changes will help to better characterize why the same genotype results in different phenotypes and, consequently, allows for developing a more nuanced picture of the interplay of pheno- and genotype. Here, we briefly outline three different epigenetic mechanisms and discuss their merits in paleoepigenomic investigations.

### 2.1. MicroRNA Silencing Activity

**Figure 1:**
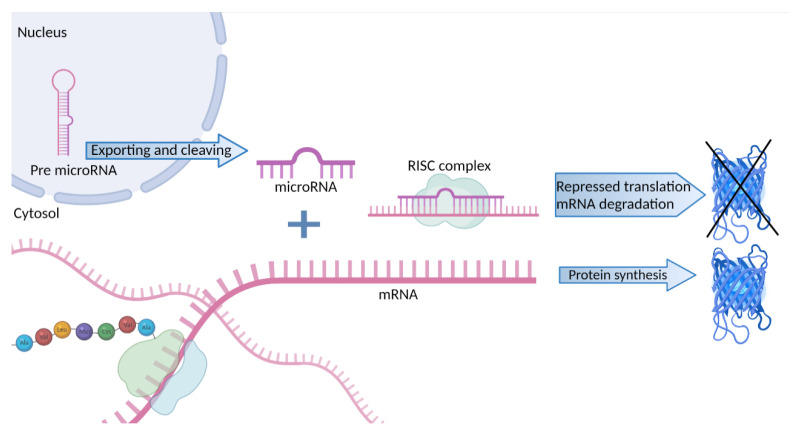
Schema of the regulation of protein synthesis through microRNA binding leading to mRNA degradation and downregulation of translational processes. Created with BioRender.com, accessed on 2 December 2021.

MicroRNAs (miRNAs) are short 15–25 nucleotide-long RNA molecules, which are responsible for post-transcriptional regulation of gene expression mainly owing to the formation of a silencing ribonucleoprotein complex called RISC (RNA-induced silencing complex) [26,27]. This mechanism is part of RNA interference, an evolutionarily old mechanism that plays a primary role in immunology [28,29]. It relies upon the binding of miRNA molecules to complementary sequences in the messenger RNA (mRNA), thereby suppressing its translation (Figure 1) and subsequently leading to the degradation of target mRNA molecules by RISC [30].

Reduced expression of miR-34a has been linked to increased lipid deposition by affecting histone H3 acetylation [31], and miR-29b influenced DNA methylation in early porcine embryo (blastocyst stage) by targeting DNA methyltransferases (DNMTs) and ten eleven translocation enzymes (TETs) [32]. Inhibition of miR-29b leads to an upregulation of *DNMT3A/B* and *TET1*, and downregulation of *TET2/3*, which increases global methylation levels and affects several genes involved in pluripotency maintenance and apoptosis regulation [32]. In addition, miR-29b influences the differentiation of human osteoclasts, highlighting its potential as a treatment for several bone diseases [33]. With the constant advancement of sequencing technologies and the recovery of shorter DNA fragments, which were traditionally excluded from sequencing efforts owing to size selection in the library preparation steps, a comprehensive description of miRNA activity is becoming more feasible and might help to further shed light on the mechanisms involved in miRNA silencing [34,35]. It has long been hypothesized that RNA molecules are too instable to persist long after a cell’s death and are thus not meaningful targets in ancient DNA (aDNA) studies. This hypothesis has been challenged as it has been shown that, under favorable preservation conditions, it is possible to identify miRNAs in ancient specimens such as the 5300 years old Tyrolean Iceman [36], with some of miRNAs still exhibiting tissue-specificity. Moreover, high-quality RNA profiles generated from a canid sample as old as 14,300 years [37] and a reanalysis of the respective RNA-seq data revealed the presence of not only common RNAs, but tissue-specific, taxonomically informative miRNA molecules [38]. These encouraging results allow us to speculate that analyses of ancient miRNAs could serve as a new approach to complement other gene expression level markers in historical, extinct specimens.

### 2.2. Histone Modifications

Histones are proteins that ensure the structural stability of the DNA molecule, enabling chromatin condensation, and thereby playing a prominent role in transcription regulation [39,40]. Several types of monomeric histone proteins bind together to form an octamer of core histones (H2A, H2B, H3, and H4) that are characterized by the presence of a histone-fold domain. This domain is crucial for maintaining chromatin integrity and DNA packaging [41]. Core histones can be replaced by variants that slightly differ in amino acid sequence composition and their incorporation changes the binding affinities of the octamer.

Histone variant H2A.Z, for example, is located at the 5’ end of gene promoters [42], marking regions of chromatin that are transcriptionally available [43]. However, depending on additional chemical modifications of the molecule, H2A.Z can be incorporated into transcriptionally repressed loci during the formation of centromeres or facultative heterochromatin [43,44,45]. Further chemical modifications of the core and variant histones include methylation, acetylation, phosphorylation, deimination (citrullination), and ubiquitination, commonly affecting histone tails. These changes play a major role in the regulation of protein–protein interactions and can, for instance, affect the chromatin structure, allowing for an immediate response to external stimuli [46,47]. One intensively studied histone modification is trimethylation of the fourth lysine in histone H3 amino acid sequence (H3K4me3; [48,49,50]). This variant is associated with the activation of promoters and, thereby, maintains the expression of, for example, *HOX* genes, which are essential for cell differentiation in early development and the formation of the body plan [51]. H3K4me3 further exemplifies the interaction of different epigenetic mechanisms in order to generate a complex regulatory network. De novo DNA methylation is performed by two methyltransferases—DNMT3A and DNMT3B, which require a homologous protein-DNMT3L that binds to histone H3. This DNMT3L–H3 complex is necessary for recruitment of DNMT3A and DNMT3B to a DNA strand and its subsequent methylation. Interestingly, H3K4 methylation inhibits the interaction between DNMT3L and H3, suppressing further recruitment of methyltransferases, a process resulting in an inverse correlation between H3K4me3 and the level of DNA methylation [52,53].

The recent technological advances in aDNA research enable us now to investigate the interplay between nucleosome structure and histone activity in extinct specimen. Based on observations of a periodic pattern in genome coverage throughout an assembly of sequencing reads, it has been suggested that such a pattern arises as a result of DNA molecules being protected from external factors by histone proteins, which results in reduced degradation [24]. As nucleosome positioning is associated with expression level [54], it could be possible to complement other paleoepigenomic evidence with nucleosome maps acting as proxies for gene expression in ancient specimens. However, more work is needed to fully comprehend the factors that influence preservation of nucleosome protection signatures [16].

### 2.3. DNA Methylation

**Figure 2:**
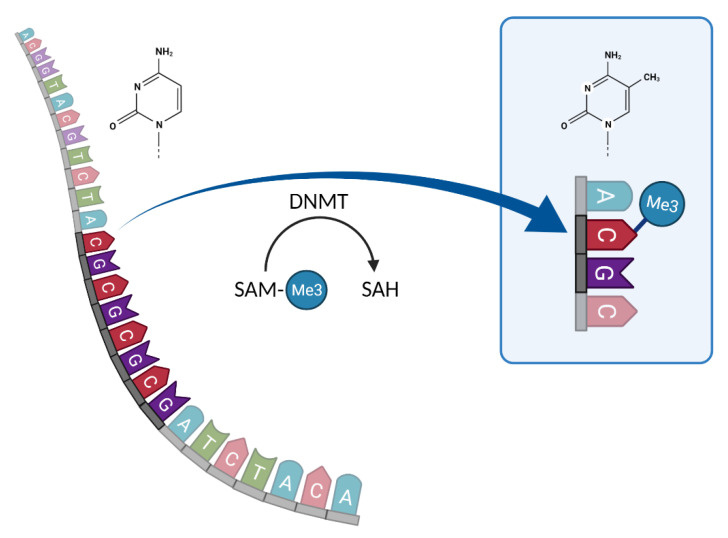
Schema of cytosine methylation by SAM in CpG positions. Created with BioRender.com, accessed on 2 December 2021.

DNA methylation is a process in which the chemical characteristics of the fifth C atom in the cytosine base of DNA are changed by DNMTs, which use S-adenosylmethionine (SAM) as a methyl group donor to transfer it onto cytosine residues (Figure 2) [55,56]. This process is usually restricted to a dinucleotide context, namely, CpG, CpA, or CpT. We will focus here on DNA methylation in the CpG context as this constitutes the majority of DNA methylation events in vertebrates [57,58]. If methylation occurs in functionally constrained regions, such as gene promoters and enhancers, it can cause changes in gene expression, leading to potential gene silencing [59,60]. Methylation has been shown to play a crucial role in X-chromosome inactivation and genomic imprinting [61,62] and is an important mechanism in embryogenesis, affecting gene function at various developmental stages with potentially lethal effects [63].

In addition, links between methylation and changes in behavior have recently been identified. In rats (*Rattus norvegicus*), the mothers’ licking, grooming, and arched-back nursing behaviors correlated with methylation patterns observed in their offspring [64]. Animals that experienced high levels of maternal care showed hypomethylation (i.e., increased gene expression) of the glucocorticoid receptor (*GR*) gene and reduced hypothalamic-pituitary-adrenal (HPA) axis response to stress compared with those individuals lacking intense maternal care. This reduced stress response was suggested to be an effect of altered interactions of *NGFI-A* transcription factor with its *GR* target sequence caused by changes in DNA methylation and histone acetylation [64]. The same mechanism of HPA stress response regulation occurs in humans and its dysfunction is associated with mental health conditions [65]. As an example, it has been proposed that suicide victims with a history of child abuse displayed hypermethylation (i.e., reduced gene expression) of *GR* compared with control groups [66]. Further evidence from schizophrenic patients indicate that sex-specific, autosomal DNA methylation patterns can be used to explain biases in psychiatric conditions [67]. Expression of DNMTs in rat hippocampi was higher after contextual fear conditioning [64,65], and inhibition of *DNMT* was associated with less freezing-behavior, a common fear response also occurring in humans [68]. Those conditioned specimens exhibited a significantly higher methylation level of *PP1*, a gene also involved in memory formation [69].

#### Effects of Environment on Methylation

A more diverse and rapidly acting methylome might open a selective advantage to organisms that need to quickly adapt to new environments. As species/populations colonize new habitats, it is commonly predicted that the genetic diversity of colonizing populations will differ from those within the source population owing to genetic drift. Evidence now suggests that changes in the methylome might outpace those at the nucleotide level [8,13].

As DNA methylation responds rather quickly to changing environments, it seems reasonable to predict that differentially methylated regions (DMRs) are preferentially located in genomic regions associated with traits that are under strong environmental constraint [70,71,72,73]. As described for the Dutch famine cohort, substantial environmental impacts on methylomes can occur in populations that differ in their diets or lifestyles [74,75,76]. Fagny and colleagues investigated this association in seven sedentary African human populations, and while three of them were traditional hunter gatherers, four populations occupied urban or rural areas and focused on farming [77]. The populations differed not only in their current lifestyle and diet, but also in their historical mode of subsistence. The analyses showed that differences in methylation are the result of different habitat, lifestyle, and diet rather than genetic ancestry. Functional investigations revealed that, for historical modes of subsistence, mainly developmental processes were enriched, and that most differences in methylation patterns between hunting and farming populations affected the immune system, including autoimmune disorders and host–pathogen interactions.

In another example, Dominguez-Salas and colleagues investigated the patterns of DNA methylation in rural Gambian populations, experiencing “hungry” and “harvest” seasons [78]. The “hungry” season coincides with the rainy season, when caloric intake from protein-based sources is strongly limited. In the “harvest” season (the dry season), a protein-based diet and hence more caloric intake is readily available. DMRs differing between children conceived during the “hungry” or during “harvest” season were located in genomic regions harboring metastable epialleles. Those metastable epialleles were in the direct vicinity of six genes (*LOC654433, EXD3, RBM46, BOLA3, ZNF678*, and *ZFYVE28),* all of which were hypermethylated in children conceived during the “hungry” season. These findings are at odds with previous results, demonstrating an overall hypomethylation in correlation with a plant-based diet [79,80,81], and thus contradicting the hypothesis that, during the “hungry” season, the diet lacks methyl group donors, leading to a decrease in methylation [82]. An alternative hypothesis is that, during the “hungry” season, maternal blood folate levels rise, possibly due to increased consumption of leafy vegetables [83] and, as folate serves as a one-carbon donor essential in the synthesis of methionine and S-adenosylmethionine [84], it might cause the observed hypermethylation.

Similar to the effect of the environment, cultural and socio-economic background was also found to influence DNA methylation, such that socio-economic position in early life influences the function of genes involved in crucial cell-signaling pathways [85,86,87], as well as in stress and inflammation-related genes [88]. Interestingly, if psychological stress can directly affect the methylome, new perspectives for the treatment and diagnosis of mental health conditions can be proposed [89]. It is noteworthy that DNA methylation signatures were observed to vary between ethnic groups. In a study performed in 573 individuals, ethnicity-related methylation signatures could not be explained by shared genetic ancestry alone, suggesting the influence of other environmental and cultural factors [90]. 

Despite these encouraging first attempts to link methylation profiles to environ-mental and social factors, more research is necessary to generate a comprehensive catalogue of environmental factors shaping the methylomes of human populations through time. Intriguingly, patterns of methylation are preserved over time [91], allowing us to investigate potential epigenetic changes associated with historical events in a species’ evolution [92]. By carefully considering the pros and cons of paleoepigenomic approaches [93], we might be able to unravel the adaptive evolution as a consequence of varying selective pressure in the past.

## 3. Paleoepigenomics

### 3.1. Ancient DNA (aDNA) Challenges

Given the rapid response of the epigenome to environmental conditions, we could speculate if historical epochs in human evolution have left a mark in the epigenome of our ancestors, allowing us to associate past changes in climate, lifestyle, and culture with epigenetics [92]. While aDNA studies go back some 40 years [94], paleoepigenomics is still in its infancy, but has already provided intriguing insights [13,18,20,21,22,23,24,93,95]. Despite all the excitement, aDNA harbors some peculiarities demanding special care and precautions when utilizing this material [96,97,98]. 

One of the major obstacles in aDNA research is contamination, which can be of ancient or modern nature. Mixtures with exogenous DNA can originate from microorganisms present in materials from which DNA is isolated or can be introduced by researchers themselves [99]. To prevent exogenous DNA from entering the analyses and thereby blurring scientific conclusions, several measures are traditionally incorporated in aDNA laboratory workflows [100]. While the effect of modern contamination is less severe for epigenetic inferences, mainly because of the lack of deaminated cytosines in modern molecules, exogenous molecules will represent a significant proportion of sequencing data. Moreover, generating high coverage and quality ancient genomes is a prerequisite of any paleoepigenomic investigation and these genomes will be of use for demography and selection inferences, in which contamination is not just a nuisance, but can dramatically affect the outcome.

After an organism’s death, both DNA and RNA undergo degradation caused by endogenous nucleases, radiation, oxidative, and hydrolytic processes [101]. DNA fragmentation is mainly caused by water exposure, which affects glycosidic bonds, resulting in depurination/depyrimidination and further abasic site formation, which facilitates strand breaks, yielding in highly fragmented aDNA molecules [102,103,104].

One special case of hydrolytic damage is the deamination of nitrous bases, a spontaneous process most noticeable in the conversion of cytosine residues into uracil or hydroxyuracil [97,98,105]. When deaminated cytosines are incorporated into the polymerase chain reaction (PCR), C–T and G–A transitions are observed [97,98,106,107]. Prior to the introduction of next generation sequencing technologies (NGS), researchers relied upon elaborate DNA cloning methods to identify such miscoding lesions [97,98], but the ease of sequencing millions of aDNA molecules now enables us to observe more comprehensive deamination patterns (e.g., affecting the endings of aDNA fragments) and deduce the sample’s authenticity [19,105].

In addition to its use in the identification of authentic ancient molecules, deamination patterns also enable us to study ancient methylomes and investigate epigenetic changes at the time of its action in the past [14,15,24,95,108]. Unmethylated cytosines are deaminated into uracils, causing CpG → UpG modifications; methylated cytosines, on the other hand, are deaminated into thymines at a faster pace [25,109], which results in CpG → TpG patterns. Several methods have been proposed to address changes in the methylation landscape in aDNA [24,109,110]. Bisulfite treatment, for instance, converts unmethylated cytosines into uracils while methylated CpGs remain intact. Uracils are subsequently sequenced as thymines, thus resulting in CpG → TpG substitutions in NGS data. The remaining CpG positions are those that were methylated in the original sequence [111]. In contrast, a uracil-DNA glycosylase (UDG) treatment introduces abasic sites in place of uracil residues, which further inhibit polymerase activity. Endonuclease VIII is subsequently used to cleave out abasic sites [112], leaving TpGs as signatures of methylated CpG. UDG treatment has an advantage over bisulfite sequencing as it does not require optimization for its application to shorter DNA fragments. 

In addition to the technological obstacles, signatures of DNA methylation are tissue-specific and DNA from ancient specimens is mostly obtained from well-preserved sources like bones, hair, and teeth, restricting generalization of the observed patterns [93]. However, many genomic regions become methylated/unmethylated in early, developmental stages and those epigenetic marks are further passed throughout developing tissues with no changes to methylation status [113,114]. Furthermore, DNA methylation maps of major human organs revealed a high similarity in methylation signatures of developmentally close tissues [115], potentially allowing to draw cross-tissue relevant conclusions. In a recent review, Smith and Non (2021) have discussed the prospects of paleoepigenomic studies and highlighted further limitations such as our inability to reach unequivocal cause–effect conclusions, or the lack of fine scale resolution of methylation estimates leaving us exclusively with differentially methylated regions instead of positions. However, future research at the interplay of epi- and functional genomics in a modern specimen coupled with a constant methodological advancement in aDNA research will help to overcome these hurdles, and eventually allow us to develop methylome maps of entire ancient populations. Lastly, bearing in mind the invasive/destructive nature of aDNA research and the historical/cultural background of any ancient human remain, we need to implement a respectful and ethical processing of the actual sample as well as interpret the results with the utmost care and sensibility [116,117,118].

### 3.2. Human Paleoepigenomic Case Studies

Paleoepigenomic research has already delivered intriguing results [16,18,92,119], in particular with regard to human evolution [15,20,21,22,24,25,120]. One of the most elaborate investigations was performed by Gokhman and colleagues, who analyzed methylation maps recovered from Denisovan and Neanderthal samples and compared those to present-day human data [15,20,21]. In their original work, the authors assigned over 1000 DMRs with respect to loci specific to Denisovans, Neanderthals, and modern humans [15]. Among the Neanderthal-specific DMRs were several hypermethylated regions within the HOXD cluster, which contains genes crucial for limb development in vertebrates [121]. Such differences possibly explain the relatively shorter limbs of Neanderthals compared with modern humans [122]. Moreover, subsequent analyses led to the successful reconstruction of morphological features of the Denisovan specimen by utilizing methylation maps [20]. The authors focused their work on DMRs that occur in well-described genomic regions associated with specific phenotypes by leveraging the Human Phenotype Ontology [123]. They used cranial and post-cranial features of Neanderthals and chimpanzees in conjunction with the respective methylation maps to infer the phenotype of a Denisovan specimen. The precision of the method, described as the percentage of predictions that match known skeletal features, reached ~83% and ~61% in Neanderthal and chimpanzees, respectively. This approach led to the identification of 56 morphological features that distinguished Denisovans from Neanderthals or modern humans, including a longer dental arch, greater facial protrusion, and increased pelvic size, and it is noteworthy that some of the inferred features matched indeed the only described Denisovan jawbone fossil [124]. Moreover, parallels exist between human-specific DMRs identified in these ancient specimens and those DMRs observed in Gambian children conceived during the “hungry” season [15,78,82]. This suggests that the effects of diet on the genome-wide pattern of methylation are readily detectable in archaic humans and that they may have experienced similar environmental conditions as populations with seasonally limited food access [92]. Lastly, Gokhman and co-workers indicated that DMRs specific to present-day humans are more often located near to or in genes related to diseases including neurological and psychiatric conditions, altogether advocating paleoepigenomic studies as a tool to investigate genome/phenome interactions with past environments [15].

### 3.3. Practical Paleoepigenomic Considerations—The Importance of Sequencing Depth

Paleoepigenomic approaches open new windows into the past, allowing us to assess gene activity in extinct specimens and, thereby, allude to environmental, health, social, or cultural circumstances shaping them [18,92]. While these are fascinating prospects, the quality of the data necessary to achieve meaningful results remains to be determined. Pioneering paleoepigenomic work by Gokhman and colleagues [15,20,21] relied upon access to high-quality archaic genomes, sequenced at an unprecedented depth [17,125,126,127], but most studies utilizing aDNA have generated complete genomes of significantly lower average coverage, barely exceeding fivefold [128,129].

As average genome-wide coverage inherently determines the final resolution of pale-oepigenomic inferences [23], we analyzed data from two previously published ancient genomes [130,131] and one that is currently finalized for publication (Fraser & Sánchez Quinto et al. [132], unpublished data, manuscript in preparation) [133] to assess the actual increase in precision with higher coverage. We used a new software package *DamMet* [23], which is a powerful extension to *epiPALEOMIX* [22]. The authors suggested that a confident estimation of local methylation patterns (represented by the estimator *f*) would require an average genome-wide coverage of at least 20×. By exploiting the three ancient genomes, we are able to propose a lower coverage that might be a better balance between cost and benefit of paleoepigenomic investigations. However, the variance in individual sample preservation, determined by its age or damage patterns, might require a more in-depth sequencing effort for additional specimens.

**Figure 3:**
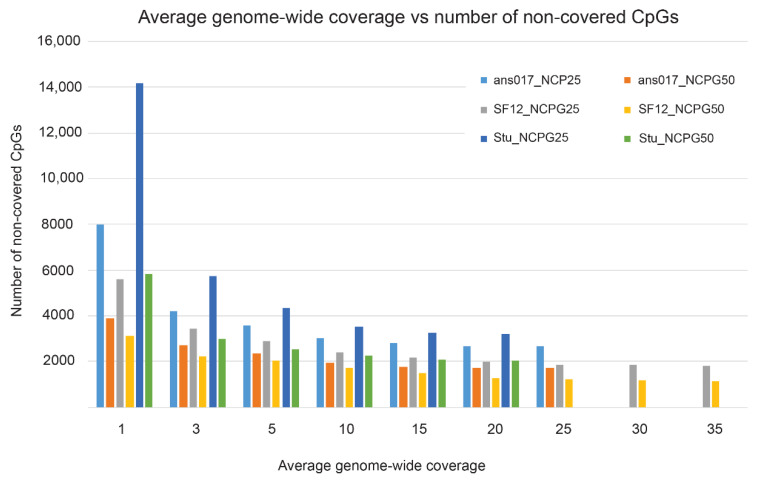
Number of non-covered CpG positions in relation to the average genome-wide coverage. Please note that the maximum average genome-wide coverages per individual are as follows: ans017—24×, SF12—38×, and Stuttgart (Stu)—19×, respectively.

We obtained raw sequencing reads for the three ancient specimens—ans017 (Fraser & Sánchez Quinto et al. [132], unpublished data, manuscript in preparation) [133], SF12 [130], and Stuttgart [131], then processed them with the *Paleomix* pipeline [134] and estimated the average genome-wide coverage as ans017 ~24×, SF12 ~38×, and Stuttgart ~19×, respectively. We performed a down-sampling experiment based on each sample’s respective full coverage, generating datasets of 1×, 3×, 5×, 10×, 15×, 20×, 25×, and 30× fold coverage using *samtools* [135]. The resulting datasets were subsequently parsed into *DamMet* and the methylation estimator (*f*) was assessed for each CpG position in chromosome 20 of the publicly available human genome (GRCh37) and corrected for C > T and A > G mutations in the respective ancient genomes. Chromosome 20 was chosen as a representation of the entire genome and contains ~700,000 CpGs. In a first step, we investigated the amount of CpGs that were covered with sequencing reads in respect to the average genome-wide coverage. The NCPG parameter described the number of CpGs downstream of the focal position and was used in *DamMet* for the calculation of positional methylation (*f*), with NCPG25 indicating 25 downstream CpGs and NCPG50 indicating 50 CpGs. As expected, the number of non-covered CpGs was inversely correlated with genome-wide coverage. An average genome-wide coverage of 1× would result in approximately 2.2 to 4.4 times more non-covered CpGs, compared with the respective sample’s maximum coverage (Figure 3). While an increase in genome coverage from 5× to 10× resulted in approximately 25% more CpGs being covered (except Stu_NCPG50, Figure 3), a genome coverage larger than 15× would only marginally increase the number of covered CpGs (<3%, ans017 and Stuttgart). An exception was sample SF12, for which generally a higher coverage was needed to reach these thresholds. While the sample is the oldest investigated here (9000 BP compared with ~3000 calBCE), a close inspection of its deamination pattern revealed an exceptional preservation, with only 15% of the terminal cytosines being deaminated, which was the lowest estimate among the three samples. As the level of deamination determines the possibility to detect methylated cytosines in ancient specimen [25,110], we suggest that the exceptional preservation of SF12 resulted in the lower detection rate of methylated cytosines and would consequently necessitate an even higher coverage to retrieve additional deaminated molecules. Notably, whereas the maximum number of non-covered CpGs represented on average only 1% of the total number of CpGs on chromosome 20, those missing positions might be of functional relevance for epigenetic processes.

**Figure 4:**
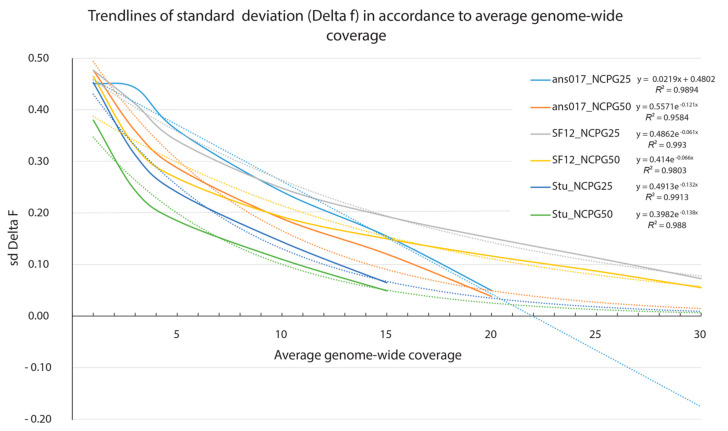
Standard deviations of Delta *f* in relation to the average genome-wide coverage. Trendlines are shown as dotted lines and the respective equations with accompanied *R*^2^ values are listed in the legend.

We next calculated the methylation estimator *f* [23] for each covered CpG. To assess the precision of *f* in relation to the average genome-wide coverage, we calculated Delta *f* (ranging from −1 to 1) as the difference in the positional *f* for each down-sampled dataset and its respective value calculated from the full dataset. As increasing the coverage should result in *f* values approaching those of the maximum coverage, and thereby increasing the precision of this estimator, Delta *f* values of 0 should become more frequent. By increasing the coverage, Delta *f* values started centering around 0, but it remained unclear at which approximate coverage an additional increase would become inefficient. To investigate this, we calculated the standard deviation of Delta *f* for each down-sampled dataset and inspected the respective trendlines. Figure 4 outlines the result of this analysis and highlights the excellent fit of the data to the regression curve indicated by R^2^ values exceeding 0.95. When assuming two times the standard deviation of the value calculated from the maximum coverage as a threshold, we concluded that, with respect to samples ans017 and Stuttgart, coverages between 10× and 15× would suffice to obtain positional *f* values close to those calculated from the maximum coverage. Sample SF12, however, would require a coverage of 20× in order to reach this threshold.

Our results demonstrate that average genome-wide coverages between 10× and 15× could suffice to perform sophisticated paleoepigenomic analyses. While such a coverage might be sufficient for many ancient specimens, individual variation does exist and should be taken into account, as shown here for sample SF12. Increasing the coverage of ancient genomes, however, comes with a high cost as sequencing experiments of aDNA harbor many pitfalls, such as a low content of endogenous DNA, high clonality, and so on. Furthermore, increasing the coverage does not linearly correspond to time and financial investments, but it rather represents an exponential correlation. Exceeding a certain coverage requires substantially more effort by involving the preparation of new extracts, new sequencing libraries, and additional sequencing itself. The suggested coverage of 10× to 15× would exceed those of standard population genetic studies utilizing ancient specimens, and such high-coverage ancient genomes could thereby be further exploited with respect to low frequency and rare variation [136], ancient pathogens [137,138], or other molecular features, allowing for further advancement of our knowledge of the molecular mechanisms that helped our ancestors to better adapt to an ever-changing environment.

## 4. Conclusions

We presented a brief overview of epigenetic research in vertebrates with a particular focus on DNA methylation in humans. Environmental conditions such as diet, lifestyle, changes in population structure, and different stressors on the epigenome are essential evolutionary mechanisms, requiring an organism to quickly adapt. Human evolution is characterized by dramatic demographic changes such as large-scale migrations, lifestyle transitions, and cultural developments, and we are now able to paint a more detailed picture of the genomic and epigenomic footprints these events have left through time. Recent investigations have started to uncover the potential of paleoepigenomics, and novel findings will not only allude to the effects of the environment on the methylomes, but might also provide a better understanding of modern lifestyle diseases.
